# Mixed infections with *Chlamydia *and porcine epidemic diarrhea virus - a new *in vitro *model of chlamydial persistence

**DOI:** 10.1186/1471-2180-10-201

**Published:** 2010-07-27

**Authors:** Nicole Borel, Claudia Dumrese, Urs Ziegler, Andrea Schifferli, Carmen Kaiser, Andreas Pospischil

**Affiliations:** 1 Institute of Veterinary Pathology, Vetsuisse Faculty, University of Zurich, Zurich, Switzerland; 2 Center for Microscopy and Image Analysis, University of Zurich, Zurich, Switzerland

## Abstract

**Background:**

Chlamydiae induce persistent infections, which have been associated with a wide range of chronic diseases in humans and animals. Mixed infections with *Chlamydia *and porcine epidemic diarrhea virus (PEDV) may result in generation of persistent chlamydial infections. To test this hypothesis, an *in vitro *model of dual infection with cell culture-adapted PEDV and *Chlamydia abortus *or *Chlamydia pecorum *in Vero cells was established.

**Results:**

Infected cultures were investigated by immunofluorescence (IF), transmission electron microscopy (TEM) and re-infection experiments. By IF, *Chlamydia*-infected cells showed normal inclusions after 39 hpi. Dual infections with *Chlamydia abortus *revealed a heterogenous mix of inclusion types including small inclusions consisting of aberrant bodies (ABs), medium-sized inclusions consisting of ABs and reticulate bodies and normal inclusions. Only aberrant inclusions were observable in dual infection experiments with *Chlamydia pecorum *and PEDV. TEM examinations of mixed infections with *Chlamydia abortus *and *Chlamydia pecorum *revealed aberrant chlamydial inclusions containing reticulate-like, pleomorphic ABs, which were up to 2 μm in diameter. No re-differentiation into elementary bodies (EBs) was detected. In re-infection experiments, co-infected cells produced fewer EBs than monoinfected cells.

**Conclusions:**

In the present study we confirm that PEDV co-infection alters the developmental cycle of member species of the family *Chlamydiaceae*, in a similar manner to other well-described persistence induction methods. Interestingly, this effect appears to be partially species-specific as *Chlamydia pecorum *appears more sensitive to PEDV co-infection than *Chlamydia abortus*, as evidenced by TEM and IF observations of a homogenous population of aberrant inclusions in PEDV - *Chlamydia pecorum *co-infections.

## Background

Chlamydiae are implicated in a wide variety of diseases in both animals and humans. Although acute infections in animal chlamydioses are the most commonly reported, chronic chlamydial infections are also associated with a variety of diseases in humans and animals. These latter infections are characterized by inflammation and scarring resulting in significant damage of the host. A causative role in chronic diseases requires that chlamydiae persist within infected tissue for extended periods of time. Current theories, based primarily on *in vitro *data, suggest that chlamydial persistence, and the resulting chronic inflammation, is linked to morphological and metabolic conversion of the actively replicating and intracellular reticulate body (RB) into an alternative, non-replicative form known as an aberrant body (AB) [1]. *In vitro*, alterations of the normal developmental cycle of *Chlamydia trachomatis *and *Chlamydia pneumoniae *can be induced by Interferon-γ (IFN-γ), tumor necrosis factor-α (TNF-α) and penicillin G exposure as well as amino acid or iron deprivation and monocyte infection [2,3]. To date, in vitro models for animal pathogens, *Chlamydia abortus *and *Chlamydia pecorum *have not been described although both organisms are associated with chronic disease in koalas and small ruminants [1].

In pigs, several chlamydial species, including *Chlamydia abortus*, *Chlamydia psittaci*, *Chlamydia pecorum *and *Chlamydia suis*, have been implicated in a variety of disease conditions including conjunctivitis, pneumonia, pericarditis, polyserositis, arthritis, abortion and infertility [4]. In the gastrointestinal tract, chlamydiae appear to be highly prevalent but only occasionally cause enteritis. They have been found in the intestine of diarrheic and healthy pigs and could be demonstrated in mixed enteric infections [5-7]. Pospischil and Wood [7] first described an association between *Chlamydiaceae *and lesions in the intestinal tract of pigs and assumed a synergistic effect in co-existence with *Salmonella typhimurium*. Further, mixed infections with *Eimeria scabra*, cryptosporidia, and porcine epidemic diarrhea virus (PEDV) have been described in the past. PEDV, a member of the family Coronaviridae, is a well-known cause of diarrhea in pigs. After the identification of PEDV in 1978 by Pensaert and Debouck [8], more than a decade passed before the virus could be adapted for propagation in cell cultures. Examination of infected Vero cell cultures by direct immunofluorescence revealed single cells with granular cytoplasmic fluorescence as well as formation of syncytia with up to 50-100 nuclei or more. Typical features of syncytial cells were growth, fusion and detachment from cell layers after they had reached a certain size [9]. Biomolecular studies revealed major genomic differences between cell culture-adapted (ca)-PEDV and wild type virus [10,11].

Cell culture model of co-infection with ca-PEDV and *Chlamydia *has been established recently [12] to investigate the interaction of ca-PEDV and *Chlamydiaceae *in mixed infections and to detect possible synergistic or additive effects of possible significance in clinical enteric disease in pigs. In that study, abnormally large chlamydial forms were observed in dually infected cell layers by immunofluorescence suggesting that ca-PEDV co-infection might alter the chlamydial developmental cycle in a manner similar to that observed during persistent infections. To confirm these initial observations, we established a cell culture model of mixed infections with *Chlamydia *and a cell culture-adapted porcine epidemic diarrhea virus (ca-PEDV) and hypothesized that this would result in the generation of persistent chlamydial forms. This data demonstrates that ca-PEDV co-infection, indeed, alters the developmental cycle of *Chlamydia pecorum *and *Chlamydia abortus *in a similar manner to other inducers of chlamydial persistence.

## Results

### Vero cells can be co-infected with *Chlamydia *and ca-PEDV

Immunofluorescence (IF) labeling was used to investigate the morphologic differences of *Chlamydia *between monoinfected and dually infected monolayers using *Chlamydia *and ca-PEDV specific antibodies. Control and mock-infected cells did not stain with either antibody.

**Figure 1:**
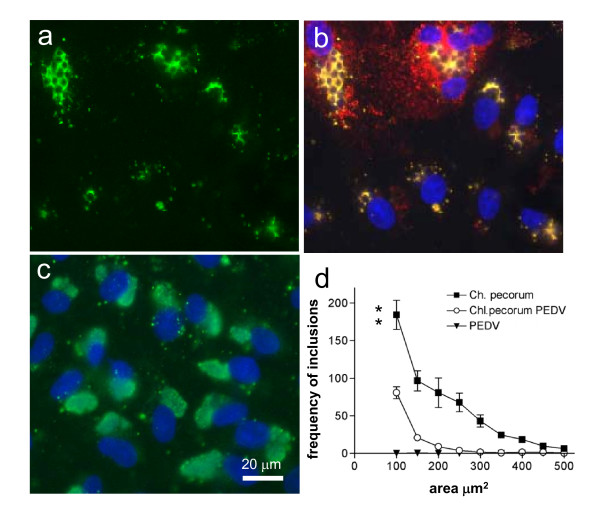
**Morphology of *Chlamydia pecorum *mono- and co-infection with PEDV**. a) Vero cells were infected with *Chlamydia pecorum *1 MOI for 39 h, with subsequent PEDV inoculation and labelled with an anti-*Chlamydia *antibody (green); b) double infected monolayer were labelled for ca-PEDV in red, *Chlamydia *in green and DNA in blue; c) *Chlamydia pecorum *mono-infected Vero cells labelled with an anti-*Chlamydia *antibody (green) and DNA staining (blue); d) Inclusion size was measured as described and the frequency of chlamydial inclusions assembled into sizes of 50 μm^2 ^area groups depicted. The difference between mono and double infected monolayers was statistically analyzed using students t-test. The groups were significantly different with p = 0.0044.

Ca-PEDV monoinfected cells showed brilliant and distinct, red cytoplasmic fluorescence. Syncytia were characterized by accumulation of nuclei in the center or the periphery of the multi-nucleated cells and moderate to bright, fine-granular, cytoplasmic ca-PEDV labeling (Figure 1b). Syncytia were categorized into small (2-15 nuclei), medium (16-30 nuclei) and large (more than 30 nuclei). In single infection experiments, syncytia at 24 h post infection were mostly large with fewer medium sized syncytia observed (data not shown). Numbers of syncytia in ca-PEDV single and dual infections were counted on the whole coverslip and mean values were determined. No difference of viral syncytia numbers for ca-PEDV monoinfection and dual infection with *Chlamydia abortus *were seen (data not shown). In contrast, numbers of viral synyctia in dual infections with *Chlamydia pecorum *were diminished compared to the respective ca-PEDV single infections (Table 1).

**Table 1:** Numbers of syncytia for ca-PEDV monoinfection compared with dual infection with *Chlamydia pecorum*

**Number of syncytia***^**a**^*			
**Experimental group***^**b**^*	**Experiment 1**	**Experiment 2**	**Experiment 3**

Mock	0	0	0

Ca-PEDV	102	23	159

*Chlamydia pecorum*	0	0	0

*Chlamydia pecorum/*ca-PEDV	8	3	16

*^a ^*Numbers of syncytia for ca-PEDV monoinfection and dual infection with *Chlamydia pecorum *were counted on the whole coverslip.

*^b ^*Vero cells were mock infected (Mock), *C. pecorum *infected, ca-PEDV infected (ca-PEDV) and *Chlamydia pecorum*/ca-PEDV co-infected as described.

**Figure 2:**
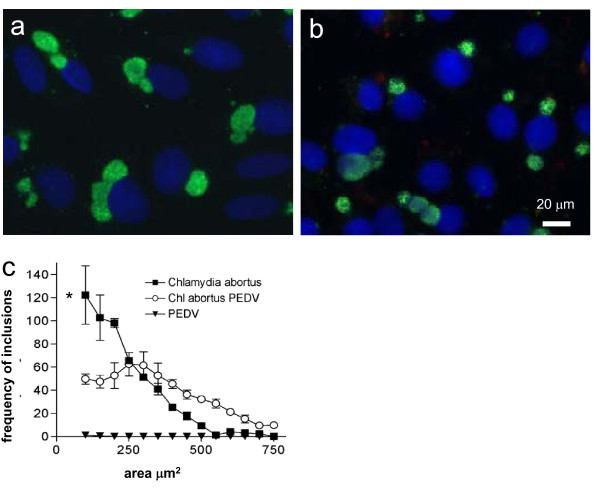
**Morphology of *Chlamydia abortus *mono- and co-infection with PEDV**. a) Vero cells were infected with *Chlamydia abortus *1 MOI for 39 h stained with an anti-*Chlamydia *antibody (green). Nuclei of Vero cells are visualized by DAPI stain (blue); b) Vero cells were infected with *Chlamydia abortus *with subsequent PEDV inoculation and stained as with an anti-*Chlamydia *antibody and DAPI; c) Frequency of inclusions with various sizes was calculated and mono and double infected cells were compared according to the inclusion size. The difference between mono and double infected monolayers was statistically analyzed using students t-test. The groups were significantly different with p = 0.0132.

IF microscopy of chlamydial single infections revealed intracytoplasmic, mainly round to ovoid, sharply outlined inclusions with brilliant, green fluorescence. *Chlamydia abortus *and *Chlamydia pecorum *infected cells had one to five, finely granular (consisting mainly of EBs) inclusion(s) per cell at 39 h post infection (Figure 1c &2a). In general, chlamydial inclusions were smaller and had more variable forms in *Chlamydia pecorum *than in *Chlamydia abortus *single infections. Infectivity was almost 100% and a moderate number of free EBs could be observed.

### Ca-PEDV co-infection alters morphology and size of chlamydial inclusions

Compared to single infections, the size and shape of chlamydial inclusions in PEDV co-infections was highly variable. In *Chlamydia abortus *co-infection experiments, three types of inclusions were observed: (i) small inclusions consisting of 1-10 aberrant bodies (ABs), (ii) medium-sized inclusions consisting of ABs and reticulate bodies (RBs), and (iii) large (normal) inclusions consisting of EBs as seen in the single infection experiments (Figure 2b).

In contrast, dual infections with ca-PEDV and *Chlamydia pecorum *resulted in the exclusive production of aberrant inclusions containing between 2-50 ABs. Chlamydial inclusions in viral syncytia grew even larger than in non-viral infected Vero cells. Overall, no normal chlamydial inclusions were observed (Figure 1a &1b).

Image analysis was used to compare inclusion size in single chlamydiae-infected Vero cells with the inclusion size in Vero monolayers that subsequently underwent ca-PEDV virus infection. To this end, inclusion size was determined in μm^2 ^and all inclusions were assembled into groups covering 50 μm^2 ^and multiples of this area.

The average frequency of *Chlamydia pecorum *inclusions between 100 μm^2 ^and 400 μm^2 ^was significantly reduced when cells were subsequently infected with ca-PEDV. In other words, *Chlamydia pecorum *inclusions were highly significant smaller in ca-PEDV dual infections than in those infections without the addition of virus (Figure 1d) as analyzed by t-test (p = 0.0044). The additional changes observed in the shape of all inclusions growing in virus-infected monolayers indicated the induction of *Chlamydia pecorum *persistence, since the finely dispersed staining reverted to grape-like structures (Figure 1a &1b).

The changes of chlamydial inclusion size by subsequent virus addition to *Chlamydia abortus *are different to those we observed in the *Chlamydia pecorum *dual infection experiments. The frequency of inclusions observed between a size range of 0-200 μm^2 ^was significantly (p = 0.0132) reduced under virus infection but the amount of medium sized and big inclusions 300 - 700 μm^2 ^was increased (Figure 2c). The morphology of *Chlamydia abortus *inclusions was also found to differ in the population when co-infected with ca-PEDV. Smaller inclusions were generally observed in aberrant shapes compared to larger inclusions, which appeared similar to normal actively growing inclusions showing finely dispersed staining (Figure 2b). This effect might be due to an incomplete induction of persistence of *Chlamydia abortus *when cells were ca-PEDV coinfected.

### Co-infection with ca-PEDV induced ultrastructural morphological changes in *Chlamydia abortus *and *Chlamydia pecorum*

Persistent forms of *Chlamydia trachomatis *and *Chlamydia pneumoniae *are well described by their characteristic electron microscopic appearance [2,13,14]. Thus, chlamydial ultrastructure in single and co-infected cells was compared by transmission electron microscopy (TEM).

**Figure 3:**
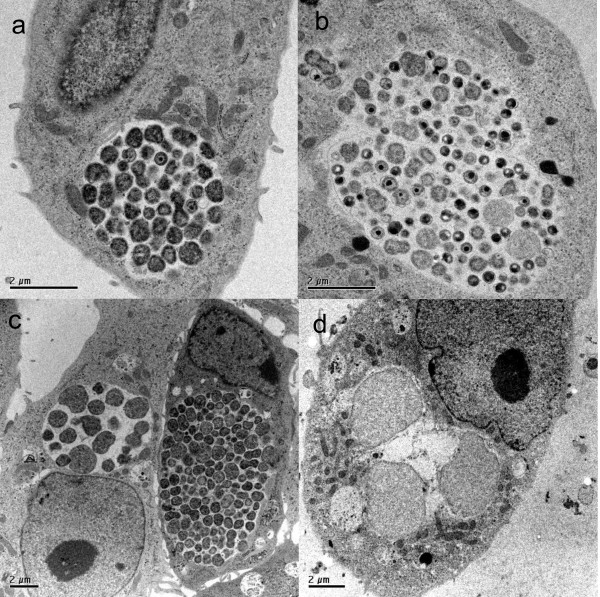
**Ultrastructure of chlamydial infection**. Vero cells were infected with *Chlamydia abortus *(MOI 1) or *Chlamydia pecorum *(MOI 1), respectively for 39 h, fixed with glutaraldehyde, and further processed as described in material and methods. a) *Chlamydia abortus *mono infection containing many RBs and a few EBs. b) A more lobular *Chlamydia pecorum *mono infection inclusion containing many RBs, IBs and EBs. c) *Chlamydia abortus *double infection with ca-PEDV showing an inclusion of the growing phenotype on the right aspect of the picture and an inclusion consisting of RBs and large aberrant bodies in the adjacent cell on the left aspect of the picture. d) *Chlamydia pecorum *double infection with ca-PEDV depicting a small inclusion with aberrant bodies exclusively.

At 24 h after viral infection, viral-induced syncytia containing vacuoles filled with viral particles were present in ca-PEDV-monoinfected and dual infections. The viral particles showed the typical Coronavirus morphology with a diameter between 50 to 130 nm (data not shown). At 39 h after chlamydial infection, large intracytoplasmic chlamydial inclusions in single infected cells could be observed in Vero cells infected with *Chlamydia abortus *or *Chlamydia pecorum. *The inclusions observed contained variable numbers of morphologically normal RBs and EBs and were generally located near the host cell nucleus, often surrounded by mitochondria (Figure 3a and 3b).

TEM examinations of mixed infections (ca-PEDV and *Chlamydia abortus *or *Chlamydia pecorum*) revealed aberrant chlamydial inclusions containing fewer bacteria than typical inclusions and were located in viral syncytia or single cells without viral infection. Aberrant inclusions consisted of reticulate-like, pleomorphic, aberrant bodies (ABs), which were in general larger in diameter (up to 2 μm) than typical reticulate bodies (RBs), with a sparse densitometric appearance and no re-differentiation into elementary bodies (EBs). As already observed in IF investigations, three types of inclusions were present in dual infections with ca-PEDV and *Chlamydia abortus *(Figure 3c), whereas dual infections with ca-PEDV and *Chlamydia pecorum *resulted in the exclusive production of aberrant inclusions consisting of 2-50 ABs (Figure 3d).

Neither chlamydial inclusions nor ca-PEDV virions were visible in mock-infected cells.

### ca-PEDV superinfection inhibition of infectious chlamydial EBs is chlamydial strain-specific

**Figure 4:**
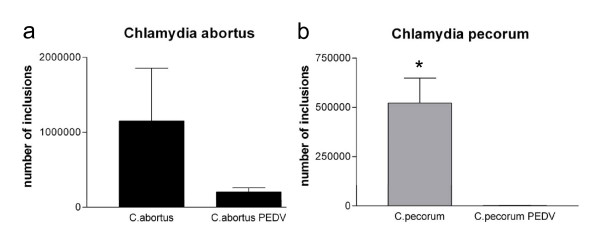
**Reinfection analysis of three independent experiments**. a) number of inclusions of *Chlamydia abortus *inclusions after reinfection from mono and double infection. b) number of inclusions of *Chlamydia pecorum *after reinfection from mono and double infection.

Previous studies have demonstrated that chlamydial persistent forms are non-infectious [2]. Reduced number or even a lack of EBs in co-infected cells in TEM suggested arrested chlamydial developmental cycle with halted maturation from RB to EB. To ascertain the effect of ca-PEDV inhibition of chlamydial EB production, the yield of infective chlamydial progeny was determined after 40 h of re-infection in three independent experiments for *Chlamydia abortus *(Figure 4a) and for *Chlamydia pecorum *(Figure 4b). Neither mock nor ca-PEDV monoinfected cells produced detectable infectious EBs, whereas *Chlamydia abortus *and *Chlamydia pecorum *single infections cells produced abundant EBs. Co-infected cells produced fewer infectious EBs than non-viral infected cells, demonstrating that production of infectious chlamydial progeny was essentially diminished by ca-PEDV-co-infection. Eradication of infectious EB production was almost complete in *Chlamydia pecorum *double infection, analyzed by reinfection experiments and found to be statistically different as analyzed by t-test (p = 0.0145) (Figure 4b). In *Chlamydia abortus *reinfection analysis, several EBs could still be observed in spite of the co-infection with ca-PEDV (Figure 4a). Statistical analysis by t-test revealed no statistical difference (p = 0.2523) presumably due to the high variation in the data.

This data is consistent with the observations from our IF and ultrastructural analysis.

### Chlamydial co-infection does alter ca-PEDV infection depending on the chlamydial species but does not alter viral ultrastructure

To determine whether chlamydial pre-infection altered subsequent viral infection, numbers of syncytia and ca-PEDV-infected cells from single and co-infected monolayers of three unrelated experiments were counted. Mock-infected and *Chlamydia *only infected cells produced no virions. The difference between virus-infected cells and co-infection with *Chlamydia abortus *was minimal. The number of syncytia detected were within the same range (data not shown) indicating that chlamydial co-infection with *Chlamydia abortus *does not alter ca-PEDV infection or the development of syncytia. In contrast, numbers of syncytia in co-infection with *Chlamydia pecorum *were reduced compared to single ca-PEDV infection (Table 1).

Overall numbers of single viral infected cells were low in both single and co-infection experiments, and no significant difference between the two chlamydial species was obvious (data not shown).

Viral morphology was also studied by TEM. In ca-PEDV single and co-infected cells, viral particles were unaltered indicating that chlamydial co-infection did not induce any changes in viral ultrastructural morphology.

## Discussion

While a previous study [12] primarily investigated the interaction of ca-PEDV and *Chlamydiaceae *in mixed infections to detect possible synergistic or additive effects of these two pathogens, questions remained about whether viral infection could potentially induce the persistent chlamydial phenotype. Enlarged chlamydial inclusions were described in that study in the ca-PEDV co-infection model with *Chlamydia abortus *and *Chlamydia pecorum *but no further ultrastructural analysis has been subsequently performed. In this study, *in vitro *models of *Chlamydia abortus *and *Chlamydia pecorum *persistence were established using co-infection with ca-PEDV. Several experimental methods were used to demonstrate the characteristic features of chlamydial persistence, including altered ultrastructural morphology and decreased production of infectious EBs. Our results demonstrated that ca-PEDV-co-infection alters the chlamydial developmental cycle similarly to other inducers of chlamydial persistence. A similar co-infection model has been recently described by Deka et al. (2006) [15]. In that study, it was shown that *Chlamydia trachomatis *enters a viable but non-cultivable, persistent state with herpes simplex virus type 2 (HSV-2) co-infected host cells. In contrast, a similar study investigating a co-infection model with *Chlamydia trachomatis *and genital mycoplasmas, *Mycoplasma genitalium *and *Mycoplasma hominis*, did not change the morphology of chlamydial RBs, indicating that co-infection of these two microorganisms is likely to be independent and not related to the onset of chlamydial persistence [16]. In the study by Deka et al. (2006) [15], HeLa monolayers were first infected with *Chlamydia trachomatis *and 24 h later with HSV-2. In our study, the optimal experimental protocol for co-infection procedure was developed, based on our own earlier study [12], and optimization experiments performed as a part of the current study (data not shown). To this end, Vero monolayers were first infected with *Chlamydia *and later with ca-PEDV, thus the suspected inducer of persistence would be introduced after chlamydial infection and differentiation into RBs. Simultaneous infection of *Chlamydia *and ca-PEDV has been performed earlier [12], but did not result in persistent infection in our preliminary experiments (data not shown) and was not considered further as interference of chlamydial infection and concurrent viral uptake could have influenced the results. Viral infection and subsequent development of syncytia was not affected by co-infection with *Chlamydia abortus *as demonstrated by unaltered numbers of syncytia observed in the co-infection experiments. In contrast, viral syncytia formation was dramatically decreased in Vero cells double infected with ca-PEDV and *Chlamydia pecorum*. If *Chlamydia pecorum *infection might induce a down regulation of the host PEDV receptor needed for syncytium formation at 14-15 hours post-chlamydial infection, this could produce a reduction in syncytium formation without reducing viral entry or replication - the possible persistence inducer mechanism.

Interestingly, chlamydial persistence was more prominent in co-infection with *Chlamydia pecorum *than with *Chlamydia abortus*, indicating possible species-specific differences. Limited reports are available for *in vitro *models of chlamydial persistence from non-*Chlamydia trachomatis *and *Chlamydia pneumonia*e strains. Kaltenboeck and Storz (1992) [17] suggested that strain 1710S of *Chlamydia pecorum *is highly nutrient dependent and this could elicit aberrant forms. Indeed, aberrant forms of this strain were significantly present in our study. Previously, only limited data have been published on persistent infection of L cells with an ovine abortion strain of *Chlamydia psittaci *(current classification: *Chlamydia abortus*) [18]. It should be noted, that in the latter study, chlamydial persistence was not demonstrated using the characteristic features now associated with the morphology of persistent chlamydial infections. Detailed description of electron microscopic observations on the effects of penicillin on the morphology of *Chlamydia psittaci *Cal10 in L cells showing aberrant chlamydial stages were published by Matsumoto and Manire [13].

The different occurence of persistent forms in co-infection with *Chlamydia abortus *and *Chlamydia pecorum*, respectively, has not been described before. Differences between persistence behaviour are already known (reviewed by Hogan et al., 2004) [1] not only between different chlamydial species but also between different serovars and strains of *Chlamydia pneumoniae *and *Chlamydia trachomatis*, respectively. The fact that *Chlamydia pecorum *strain 1710S is an original swine isolate whereas *Chlamydia abortus *strain S26/3 originates from a sheep abortion and, thus, from another animal species could have an impact but needs further investigation.

The mechanism by which ca-PEDV interferes with chlamydial developmental cycle and chlamydial persistence is still unclear. It is known that Vero cells, a monkey kidney epithelial cell line, is deficient for Interferon production [19]; thus, this cytokine group well known to be capable of inducing *in vitro *persistence in *Chlamydia pneumoniae *[1], cannot be relevant for our co-infection persistence model. Co-infection experiments with ca-PEDV are best performed with Vero cells, as they have been shown to be permissive for viral replication in contrast to other cell lines such as PD5, PK 15, and HRT18 cell lines [9]. Specific measurements of primate cytokines in our co-infection model are planned in the future to elucidate the mechanism leading to chlamydial persistence. The Herpes simplex virus (HSV) co-induced *Chlamydia trachomatis *persistence model [15] has been recently been shown not to be mediated by any known persistence inducer or anti-chlamydial pathway recently [20,21]. Instead, it was hypothesized by the authors that HSV-2 attachment and/or entry into the host cell is sufficient for stimulating chlamydial persistence, suggesting a potential novel host signaling pathway could be responsible for inducing chlamydial persistence. A very recent publication by the same group showed that HSV replication is not necessary for persistence induction and that chlamydial activity could be recovered after co-infection with UV-inactivated HSV-2. Finally, it was concluded that the interaction of HSV glycoprotein D with the host cell surface is crucial to trigger chlamydial persistence [22].

Female genital tract infection often has a complex etiology, where *Chlamydia trachomatis *is present together with one or more genital agents. Epidemiological and clinical studies have shown that double infection with HSV-2 and *Chlamydia trachomatis *occurs *in vivo*; thus, the *in vitro *model described by Deka et al. (2006) [15] represents a realistic situation in human medicine. Similarities exist to the *in vitro *model established in this study as simultaneous intestinal infection with different pathogens is possible in swine *in vivo*. A recent study [23] documented the occurrence of aberrant chlamydial bodies *in vivo *in intestinal tissues of pigs. In this study, aberrant bodies of *Chlamydia suis *were demonstrated and characterized in the gut of pigs experimentally infected with *Salmonella typhimurium *by transmission electron microscopy. It was concluded by Pospischil et al. [23] that aberrant bodies occur *in vivo *in pigs and that the gnotobiotic pig model might be suitable for the study of chlamydial persistence *in vivo*.

Available intestinal tissues from experimentally infected gnotobiotic piglets (single infection and co-infection with *Chlamydia *and ca-PEDV, respectively) will be investigated in the future with the aim of further characterization of ABs *in vivo*. Although chronic chlamydial diseases in animals are of economic impact, the pig model may also reveal the important link between persistence *in vitro *and *in vivo *and, thus, help to elucidate mechanisms of chronic human chlamydial infections in the future.

## Conclusions

The present study reports a new persistence model of *Chlamydia *in co-infection with porcine epidemic diarrhea virus (PEDV). PEDV-co-infection altered the chlamydial developmental cycle similarly to other known inducers of chlamydial persistence. This new animal model could provide the important link between persistence *in vitro *and *in vivo *and, thus, would help to elucidate mechanisms of chronic human chlamydial infections in the future.

## Methods

### Media and cells

Growth medium (GM) for normal cell propagation was Minimal Essential Medium (MEM) with Earle's salts, 25 mM HEPES, without L-Glutamine (GIBCO, Invitrogen, Carlsbad, CA) and supplemented with 10% fetal calf serum (FCS) (BioConcept, Allschwil, Switzerland), 4 mM GlutaMAX-I (200 mM, GIBCO) and 0.2 mg/ml gentamycin (50 mg/ml, GIBCO).

GM without gentamycin was used for the propagation of cells for infection experiments. Infection medium was prepared as GM but without gentamycin and FCS, and was used for the infection and for the 24 h incubation period after the infection with ca-PEDV, respectively. Incubation medium was prepared as GM without gentamycin, freshly supplemented with 1 μg/ml cycloheximide (Sigma, Buchs SG, Switzerland), and used after an infection for estimation of the chlamydial titer (IFU determination).

Vero 76 cells (African green monkey kidney cells, CRL 1587 American Type Culture Collection) were seeded on round plastic coverslips (13 mm diameter, Bibby Sterilin, Stone, UK) and cultured in GM without gentamycin at 37°C until they reached confluence. Before inoculation, the cells were washed once with phosphate buffered saline (PBS).

### Chlamydial strains

Two different chlamydial strains of *Chlamydiaceae *were used in this study: *Chlamydia abortus *S26/3 (ovine abortion strain, kindly donated by Dr. G.E. Jones, Moredun Research Institute, Edinburgh, GB) and *Chlamydia pecorum *1710S (intestinal swine isolate, kindly provided by Prof. J. Storz, Baton Rouge, Louisiana, LA, USA). For initial culturing, chlamydial strains were cultured in embryonated chicken eggs, and yolk sac material was harvested, diluted 1:2 in sucrose-phosphate-glutamate (SPG) medium and stored at -80°C. Yolk sac-derived chlamydiae were then propagated in HEp-2 cell (ATCC CCL-23) monolayers and elementary bodies (EBs) were harvested and purified by disruption of HEp-2 cell monolayers with a cell scraper, sonication and centrifugation over a renografin density gradient as described elsewhere [24]. EB suspensions were stored in sucrose-phosphate-glutamic acid buffer at -80°C, after which viable titers were established using standard methods. MOI of 1 was used for chlamydial monoinfection and mixed infection, respectively.

### PEDV

Ca-PEDV strain CV777 (kindly provided by Prof. Dr. M. Ackermann, Institute of Virology, University of Zurich) was propagated as previously described [9]. The virus (10^5,5 ^TCID_50_/ml) was used undiluted (1 ml 10^5,5 ^TCID_50_/ml).

### Co-infection experimental design

Vero cells, an African green monkey kidney cell line (ATCC CRL 1587), were used for all infection experiments. They were propagated in GM without gentamycin at 37°C in an atmosphere of 5% CO_2_. Vero cells were divided into four groups: for mock infection, chlamydial infection, ca-PEDV infection, and both *Chlamydia *and ca-PEDV double infection. Host cells were infected with a MOI of 1 for *Chlamydia *and an infective dose of 1 × 10^5,5 ^TCID_50_/ml for ca-PEDV, respectively. For ca-PEDV monoinfections and negative controls, infection medium was used.

All co-infection experiments were done three times and monoinfections with *Chlamydia *and ca-PEDV were performed simultaneously. The optimal experimental protocol (adding the virus several hours after chlamydial infection) for co-infection procedure was developed before (data not shown).

For dual infections, cell monolayers were first infected with *Chlamydia *at a MOI of 1. All coverslips were centrifuged at 1000 × g for 1 h at 25°C. Timepoint 0 (T_0_) was defined after centrifugation and supernatant was replaced subsequently by incubation medium. Infected monolayers were then incubated for 14 h at 37°C (T_0 _- T_14_). All cell layers for dual infections or ca-PEDV monoinfection were exposed to a ca-PEDV suspension (1 × 10^5,5 ^TCID_50_), the samples were centrifuged again for 1000 × g for 1 h at 25°C and incubated for 24 h at 37°C. After this incubation period, all monolayers were fixed and further investigated by indirect immunofluorescence and transmission electron microscopy. Re-infection experiments were performed to compare the production of infectious chlamydial elementary bodies (EBs) between monoinfections and mixed infections.

### Indirect Immunofluorescence

For indirect immunofluorescence analyses, infected cells were fixed in absolute methanol (-20°C) for 10 min. and IF labeling of cell cultures was performed immediately after fixation. For viral antigen detection, a mouse monoclonal antibody against the M protein of PEDV (mcAb 204, kindly provided by Prof. Dr. M. Ackermann, Institute of Virology, University of Zurich), diluted 1:4 in PBS supplemented with BSA, and an Alexa Fluor 594-conjugated secondary antibody (goat anti-mouse, 1:500, Molecular Probes, Eugene, USA) were used. Chlamydial inclusions were labeled with a *Chlamydiaceae *family-specific mouse monoclonal antibody directed against the chlamydial lipopolysaccharide (mLPS; Clone ACI-P, Progen, Heidelberg, Germany) and a secondary Alexa Fluor 488-conjugated secondary antibody (goat anti-mouse, 1:500, Molecular Probes). DNA was labeled with 1 μg/ml 4', 6-Diamidin-2'-phenylindoldihydrochlorid (DAPI, Molecular Probes). All staining procedures were conducted at room temperature. Antibody incubations were carried out for 1 h (primary antibodies) or 45 min (secondary antibodies), respectively, with three washes of PBS following fixation, between and after applications of the different antibodies. Dually infected cell layers were stained using sequential double immunofluorescence labeling. Uninfected Vero cells were used as a negative control. Coverslips were mounted with Immumount (Shandon, Pittsburgh, USA) on glass slides and investigated using a Leica fluorescence microscope.

### Transmission electron microscopy

Coverslips from all experimental conditions were fixed in 2.5% glutaraldehyde (Electron Microscopy Sciences, Ft. Washington, USA) for 1-2 h, and processed by routine methods for embedding in epoxy resin (Fluka). Appropriate areas for ultrastructural investigation were selected using semithin sections (1 μm) stained with toluidine blue (Fluka, Buchs SG, Switzerland). Ultrathin sections (80 nm) were mounted on gold grids (Merck Eurolab AG, Dietlikon, Switzerland), contrasted with uranyl acetate dihydrate (Fluka) and lead citrate (lead nitrate and tri-natrium dihydrate; Merck Eurolab AG) and investigated in a Philips CM10 electron microscope.

### Chlamydial titration by subpassage

At 39 h after chlamydial infection, monolayers were scraped into 1 ml of cold infection medium, pelleted and resuspended in 1 ml of fresh medium. Infected host cells were lysed by sonication and centrifuged (500 g for 5 min) to remove pellet cell debris. Supernatants were centrifuged once (4,000 g for 60 min). Final EB pellets were resuspended in 200 μl of SPG and used to infect Vero cells plated on glass coverslips in duplicate in dilution series. All coverslips were centrifuged at 1000 × g for 1 h at 25°C. After centrifugation, the Vero cells were refed with medium containing 1 μg/ml cycloheximide and subsequently incubated for 40 h at 37°C. Fixation and staining of *Chlamydia*, ca-PEDV and DNA was performed as described above. The number of inclusions in 20 random microscopic fields per sample was determined using a Leica fluorescence microscope at a magnification of 200 ×. Duplicate coverslips were counted and the counts were averaged. The number of inclusion-forming units (IFU) in the indiluted inoculum was then calculated and expressed as IFU per 10^6 ^cells as described by Deka et al., 2006 [15].

### Imaging and statistical analyses

From duplicate samples of three independent experiments uniform random sampled images were acquired using a widefield microscope (Leica LX, Leica Microsystems Mannheim, Germany). Cells and inclusions were automatically detected according to size, shape and intensity and counted using Imaris (Bitplane AG, Zürich Switzerland).

## Competing interests

The authors declare that they have no competing interests.

## Authors' contributions

NB conceived of the study, planned the experiments, and drafted the manuscript. CD and UZ performed the imaging and statistical analyses. AS and CK carried out the cell culture experiments including immunofluorescence and transmission electron microscopy. AP participated in the design and coordination of the study and helped to draft the manuscript. All authors read and approved the final manuscript.

## References

[B1] Hogan RJ Mathews SA Mukhopadhyay S Summersgill JT Timms P Chlamydial persistence: beyond the biphasic paradigm Infect Immun 2004 72 1843 1855 10.1128/IAI.72.4.1843-1855.2004 15039303 PMC375192

[B2] Beatty WL Morrison RP Byrne GI Persistent chlamydiae: from cell culture to a paradigm for chlamydial pathogenesis Microbiol Rev 1993 58 686 699 10.1128/mr.58.4.686-699.1994 PMC372987 7854252

[B3] Beatty WL Byrne GI Morrison RP Morphologic and antigenic characterization of interferon gamma-mediated persistent *Chlamydia trachomatis *infection *in vitro* Proc Natl Acad Sci USA 2003 90 3998 4002 10.1073/pnas.90.9.3998 PMC46433 8387206

[B4] Taylor DJ Straw BE, Allaire SD, Mengeling WL, Taylor DJ Chlamydiae Diseases of Swine 1999 8 Iowa State University Press, Ames, Iowa 619 624

[B5] Nietfeld JC Leslie-Steen P Zeman DH Nelson D Prevalence of intestinal chlamydial infection in pigs in the midwest, as determined by immunoperoxidase staining Am J Vet Res 1997 58 260 264 9055971

[B6] Szeredi L Schiller I Sydler T Guscetti F Heinen E Corboz L Eggenberger E Jones GE Pospischil A Intestinal *Chlamydia *in finishing pigs Vet Pathol 1996 33 369 374 10.1177/030098589603300401 8817833

[B7] Pospischil A Wood RL Intestinal *Chlamydia *in pigs Vet Pathol 1987 24 568 570 3331859 10.1177/030098588702400617

[B8] Pensaert MB Debouck P A new coronavirus-like particle associated with diarrhea in swine Arch Virol 1978 58 243 247 10.1007/BF01317606 83132 PMC7086830

[B9] Hofmann M Wyler R Propagation of the virus of porcine epidemic diarrhea in cell culture J Clin Microbiol 1988 26 2235 2239 2853174 10.1128/jcm.26.11.2235-2239.1988 PMC266866

[B10] Duarte M Tobler K Bridgen A Rasschaert D Ackermann M Laude H Sequence analysis of the porcine epidemic diarrhea virus genome between the nucleocapsid and spike protein genes reveals a polymorphic ORF Virology 1994 198 466 476 10.1006/viro.1994.1058 8291230 PMC7131309

[B11] Tobler K Ackermann M Talbot PJ, Levy GA PEDV leader sequence and junction sites Corona and related viruses 1994 Plenum Press, New York 541 542

[B12] Stuedli A Grest P Schiller I Pospischil A Mixed infections *in vitro *with different *Chlamydiaceae *strains and a cell culture adapted porcine epidemic diarrhea virus Vet Microbiol 2005 106 209 223 10.1016/j.vetmic.2004.10.023 15778027 PMC7126122

[B13] Matsumoto A Manire GP Electron microscopic observations on the effects of penicillin on the morphology of *Chlamydia psittaci* J Bacteriol 1970 101 278 285 5413965 10.1128/jb.101.1.278-285.1970 PMC250478

[B14] Byrne GI Ouellette SP Wang Z Rao JP Lu L Beatty WL Hudson AP *Chlamydia pneumoniae *expresses genes required for DNA replication but not cytokinesis during persistent infection of HEp-2 cells Infect Immun 2001 69 5423 9 10.1128/IAI.69.9.5423-5429.2001 11500413 PMC98653

[B15] Deka S Vanover J Dessus-Babus S Whittimore J Howett MK Wyrick PB Schoborg RV *Chlamydia trachomatis *enters a viable but non-cultivable (persistent) state within herpes simplex virus type 2 (HSV-2) co-infected host cells Cell Microbiol 2006 8 149 162 10.1111/j.1462-5822.2005.00608.x 16367874

[B16] Baczynska A Birkelund S Christiansen G Chernesky M, Caldwell H, Christiansen G, Clarke IN, Kaltenboeck B, Knirsch C, Kuo CC, Mahony J, Rank RG, Saikku P, Schachter J, Stamm WE, Stephens RS, Summersgill JT, Timms P, Wyrick PB *Chlamydia trachomatis *and genital mycoplasmas in the co-infection model - *in vitro *study Proceedings of the Eleventh International Symposium on Human Chlamydial Infections: 18-23 June 2006; Niagara-on-the-Lake, Ontario, Canada 2006 International Chlamydia Symposium, San Francisco, CA 225 228

[B17] Kaltenboeck B Storz J Biological properties and genetic analysis of the *omp A *locus in *chlamydiae *isolated from swine Am J Vet Res 1992 53 1482 1487 1358014

[B18] Perez-Martinez JA Storz J Persistent infection of L cells with an ovine abortion strain of *Chlamydia psittaci* Infect Immun 1985 50 453 8 4055027 10.1128/iai.50.2.453-458.1985 PMC261974

[B19] Chew T Noyce R Collins SE Hancock MH Mossman KL Characterization of the interferon regulatory factor 3-mediated antiviral response in a cell line deficient for IFN production Mol Immunol 2009 46 393 9 2009 10.1016/j.molimm.2008.10.010 19038458

[B20] Deka S Vanover J Sun J Kintner J Whittimore J Schoborg RV An early event in the herpes simplex virus type-2 replication cycle is sufficient to induce *Chlamydia trachomatis *persistence Cell Microbiol 2007 9 725 37 10.1111/j.1462-5822.2006.00823.x 17140408

[B21] Vanover J Sun J Deka S Kintner J Duffourc MM Schoborg RV Herpes simplex virus co-infection-induced *Chlamydia trachomatis *persistence is not mediated by any known persistence inducer or anti-chlamydial pathway Microbiology 2008 154 971 8 10.1099/mic.0.2007/012161-0 18310043

[B22] Vanover J Kintner J Whittimore J Schoborg RV Interaction of HSV-2 glycoprotein D with the host cell surface is sufficient to induce *Chlamydia trachomatis *persistence Microbiology 2010 in press 20110302 10.1099/mic.0.036566-0 PMC2889450

[B23] Pospischil A Borel N Chowdhury EH Guscetti F Aberrant chlamydial developmental stages in the gastrointestinal tract of pigs spontaneously and experimentally infected with *Chlamydia suis* Vet Microbiol 2009 135 147 56 10.1016/j.vetmic.2008.09.035 18950970

[B24] Howard L Orenstein NS King NW Purification on renografin density gradients of *Chlamydia trachomatis *grown in the yolk sac of eggs Appl Microbiol 1974 27 102 106 4855645 10.1128/am.27.1.102-106.1974 PMC379975

